# Cost of chiropractic versus medical management of adults with spine-related musculoskeletal pain: a systematic review

**DOI:** 10.1186/s12998-024-00533-4

**Published:** 2024-03-06

**Authors:** Ronald Farabaugh, Cheryl Hawk, Dave Taylor, Clinton Daniels, Claire Noll, Mike Schneider, John McGowan, Wayne Whalen, Ron Wilcox, Richard Sarnat, Leonard Suiter, James Whedon

**Affiliations:** 1 American Chiropractic Association, 2008 St. Johns Avenue, Highland Park, Illiois. 60035, Arlington, VA USA; 2 Texas Chiropractic College, 5912 Spencer Highway, Pasadena, TX 77505 USA; 3 VA Puget Sound Health Care System, 9600 Veterans Drive Southwest Tacoma, Tacoma, WA 98493-0003 USA; 4 University of Pittsburgh, 100 Technology Drive, Suite 500, Pittsburgh, PA 15219 USA; 5 Saint Louis University, 3674 Lindell Blvd, St. Louis, MO 63108 USA; 6 Clinical Compass-Past Chairman, 9570 Cuyamaca St Ste 101, Santee, CA 92071 USA; 7 Private Practice, 204 Pinehurst Dr. SW, Suite 103, Tumwater, 9850 USA; 8 LP AMI Group, AMI Group, LP; 2008 St. Johns Avenue, Highland Park, IL 60035 USA; 9 Southern California University of Health Sciences, 16200 Amber Valley Drive, Whittier, CA 90604 USA

**Keywords:** Chiropractic, Conservative care, Healthcare costs, Healthcare utilization, Low back pain, Manipulation, Spinal, Opioids

## Abstract

**Background:**

The cost of spine-related pain in the United States is estimated at $134.5 billion. Spinal pain patients have multiple options when choosing healthcare providers, resulting in variable costs. Escalation of costs occurs when downstream costs are added to episode costs of care. The purpose of this review was to compare costs of chiropractic and medical management of patients with spine-related pain.

**Methods:**

A Medline search was conducted from inception through October 31, 2022, for cost data on U.S. adults treated for spine-related pain. The search included economic studies, randomized controlled trials and observational studies**.** All studies were independently evaluated for quality and risk of bias by 3 investigators and data extraction was performed by 3 investigators.

**Results:**

The literature search found 2256 citations, of which 93 full-text articles were screened for eligibility. Forty-four studies were included in the review, including 26 cohort studies, 17 cost studies and 1 randomized controlled trial. All included studies were rated as high or acceptable quality. Spinal pain patients who consulted chiropractors as first providers needed fewer opioid prescriptions, surgeries, hospitalizations, emergency department visits, specialist referrals and injection procedures.

**Conclusion:**

Patients with spine-related musculoskeletal pain who consulted a chiropractor as their initial provider incurred substantially decreased downstream healthcare services and associated costs, resulting in lower overall healthcare costs compared with medical management. The included studies were limited to mostly retrospective cohorts of large databases. Given the consistency of outcomes reported, further investigation with higher-level designs is warranted.

**Supplementary Information:**

The online version contains supplementary material available at 10.1186/s12998-024-00533-4.

## Introduction

Spine-related musculoskeletal pain is the leading cause of disability worldwide and one of the most common reasons for missed work [1]. In the United States (U.S.), healthcare costs for low back and neck pain are rising and as of 2016 were the highest for any condition, with an estimated $134.5 billion for care related to spinal pain [2].

There are many options for treatment of acute or chronic spine-related pain. These range from conservative therapies, such as manual or behavioral therapies, to medications, injection procedures and surgery [3, 4]. Approaches to management of spine-related musculoskeletal pain differ by type of provider, such as chiropractors, physical therapists, primary care medical physicians and medical specialists such as orthopedists and neurologists [5]. In the U.S., chiropractic care is one of the most commonly utilized approaches to treatment of spine-related musculoskeletal pain [6]. Chiropractic care guidelines are concordant with the American College of Physicians’ recommendations for initial management of low back pain (LBP) using non-pharmaceutical treatment [7, 8].

In the midst of rising healthcare costs, it is important to examine not only clinical outcomes but also the cost of intervention strategies for spine-related pain. Although most cases of spine-related musculoskeletal pain can be effectively managed with conservative guideline-concordant non-pharmacological and non-invasive approaches, frequently a patient’s course of care is unnecessarily escalated by use of more invasive, hazardous, and/or costly procedures [9]. The escalation of care for spine-related musculoskeletal pain may include emergency department visits, medical specialist visits, diagnostic imaging, hospitalization, surgery, interventional pain medicine techniques, prescription of drugs with high risk for addiction or abuse, and encounters for complications of spine care (e.g., adverse drug events) [9]. The escalation of spine-related musculoskeletal pain management is closely associated with increased downstream costs.

Gold et al. defined “downstream” costs as those that “may have changed, intentionally or unintentionally, as a result of the implementation strategy and intervention.”[10]^p.3^ Downstream costs may include those associated with healthcare utilization, patient and caregiver costs, productivity costs and costs to other sectors. For spine-related musculoskeletal pain, most often LBP, an emerging body of evidence suggests that downstream costs are significantly affected by the specialty of the initiating care provider [5]. Such costs typically include diagnostic tests, particularly advanced imaging [11], surgery, specialist care and medication use [12].

*The opioid epidemic.* For patients with spine-related musculoskeletal disorders, among the most important escalations of care associated with downstream human and societal costs that are receiving recent attention are opioid use, abuse and overdose. In 2017, the U.S. government declared the opioid epidemic to be a Public Health Emergency [13]. This epidemic is still on the rise, with drug overdose deaths increased by 31% in a single year, 2019–2020 [14].

It is not certain which combination of provider and/or therapy offers the most cost-effective approach to managing spine-related musculoskeletal pain. A 2015 systematic review compared the costs of chiropractic care to those of other types of health care [15]. The costs were generally lower when musculoskeletal spine care was managed by chiropractors, though the included studies contained methodological limitations [15]. The purpose of this review was to update, summarize, and evaluate the evidence for the cost of chiropractic care compared to conventional medical care for management of spine-related musculoskeletal pain [15].

## Methods

Our team followed the Preferred Reporting Items for Systematic Reviews and Meta-Analyses (PRISMA) protocol to conduct the review and registered it with PROSPERO in December 2022 prior to data abstraction (CRD42022383145). We elected a priori not to pursue meta-analysis due to heterogeneity of the included studies. Most of the included studies are cohort studies which by their nature can only show associations, cannot prove causation, and are of a lower level of evidence than RCTs, which are the study design usually included in meta-analyses. We conducted the searches and quality assessments from July through December 2022 and data abstraction from January through March 2023. The primary aim of our systematic review was to address the research question: Is chiropractic management of spine-related musculoskeletal pain in U.S. adults associated with lower overall healthcare costs as compared to medical care?

To answer the research question, we formulated PICO elements (Population, Intervention, Comparison, Outcome) as follows:

P: U.S. adults with spine-related musculoskeletal pain

I: Chiropractic management

C: Medical care

O: Healthcare costs and use of procedures estimated to increase downstream costs involved in escalation of care

Costs in a controlled setting are not often comparable to usual and customary costs in a clinical setting [16]. Therefore, in addition to randomized controlled trials, we also included economic and cohort studies that collected data specifically on cost, whether or not treatment outcomes were considered.

### Eligibility criteria

#### Inclusion criteria

- Published in peer-reviewed journal and available in Medline from inception through 10/31/2022
- English language
- Study population comprised of US adults treated for spine-related musculoskeletal pain
- Compared chiropractic management to medical care
- Cost data for treatment of spine-related musculoskeletal pain were provided
- Designs were randomized controlled trial, cohort study or economic evaluation.

#### Exclusion criteria

- Reviews, commentaries, abstracts from conference proceedings, theses, cross-sectional descriptive surveys and gray literature.
- Systematic reviews were not used as part of quality assessment or data abstraction. They were retrieved only to identify eligible studies which were not found in the literature search.
- Studies with clinical effectiveness outcomes only and no inclusion of cost or utilization data

### Literature search

We developed a search strategy based on the PICO terms, with a health sciences librarian working with the other investigators. We made several “trial runs” to refine the strategy to be sure it was as inclusive as possible while screening out obviously non-relevant citations. Our search was conducted exclusively in Medline, as relevant high-quality articles were more likely to be found in journals indexed there. We developed a search strategy with keyword clusters based on our PICO. Most publications about spine-related pain study adults (our P) and “adult” was not helpful as a limiter. Intervention (I) cluster terms were selected from a previously published search string of complementary and alternative medicine terms [17]. The Outcome (O) cluster started with terms used in a prior cost-effectiveness study [18], with the subsequent addition of indexing terms found during early search trial runs. The MeSH heading Cost-Effectiveness Analysis was not yet available at the time of our search. The complete search strategy is shown in Additional File 1.

Retrieved citations were downloaded into an EndNote library (v. 20). Using Rayyan https://rayyan.ai/, [19] online systematic review software, at least two investigators screened titles and abstracts for eligibility and resolved disagreements by discussion. At least two investigators checked the references included in all relevant systematic reviews found in the literature search and added any eligible citations not identified in our literature search to the library. At least two investigators did full-text screening of the titles remaining after title/abstract screening was completed, and disagreements were again resolved by discussion. All authors contributed during the process in review of eligible citations.

### Evaluation of risk of bias

We evaluated randomized controlled trials (RCTs) using a checklist modeled after those of the Scottish Intercollegiate Guideline Network (SIGN) [20], which we have used elsewhere [3]. An article was rated as “high quality, low risk of bias,” “acceptable quality, moderate risk of bias,” “low quality, high risk of bias,” or “unacceptable” quality. For studies analyzing treatment costs (e.g., economic studies), we developed a checklist with similar format to those of SIGN checklists [20].

For cohort studies, it was difficult to identify a single appropriate checklist because most seemed designed to be more appropriate to assess prospective cohort studies, and the most recent relevant studies related to our topic are retrospective cohort studies using large datasets. We therefore developed a checklist for prospective cohort studies after reviewing other existing checklists [20]. For retrospective or cross-sectional cohort studies, we developed a checklist combining some features of the SIGN checklist for cohort studies [20] and the NIH tool for observational cohort and cross-sectional studies [21]. These checklists included items assessing comparability of the included cohort groups, as part of the risk of bias assessment. Three investigators (RF, CH and JW), one of whom is an author of a number of cohort studies, piloted and then refined this form with a sample of studies.

Two or more investigators rated each article. Disagreements were resolved by including additional reviewers and discussing differences in ratings until they reached agreement.

Because of the large number of cohort studies, which are considered to have an inherent risk of bias due to their design, we only included studies which the investigators agreed were at minimum “acceptable quality, moderate risk of bias” using the SIGN quality criteria. We excluded any studies that the investigators found to be “low quality, high risk of bias” or “unacceptable quality.”

### Data extraction

Because it has been found that data extraction errors are frequent in systematic reviews, we followed the recommendations on data extraction in a review of data extraction guidelines and methods [22]. Before starting the process, we listed all the items we thought were necessary for answering our research question. Then we drafted a data extraction form with these items and two investigators (RF and CH) piloted it on a sample of studies. We then provided brief, online training on use of the forms with the 3 investigators who did the data extraction (RF, CH, DT). This included instructions on how disagreements would be resolved, which was to recheck the source paper and provide it to the other reviewer(s). Two investigators (RF and CH) did independent parallel extraction for all studies, and DT then reviewed the drafted tables; thus the data extraction was triple-checked. We did not attempt to subcategorize patient populations from the included studies.

## Results

**Fig. 1:**
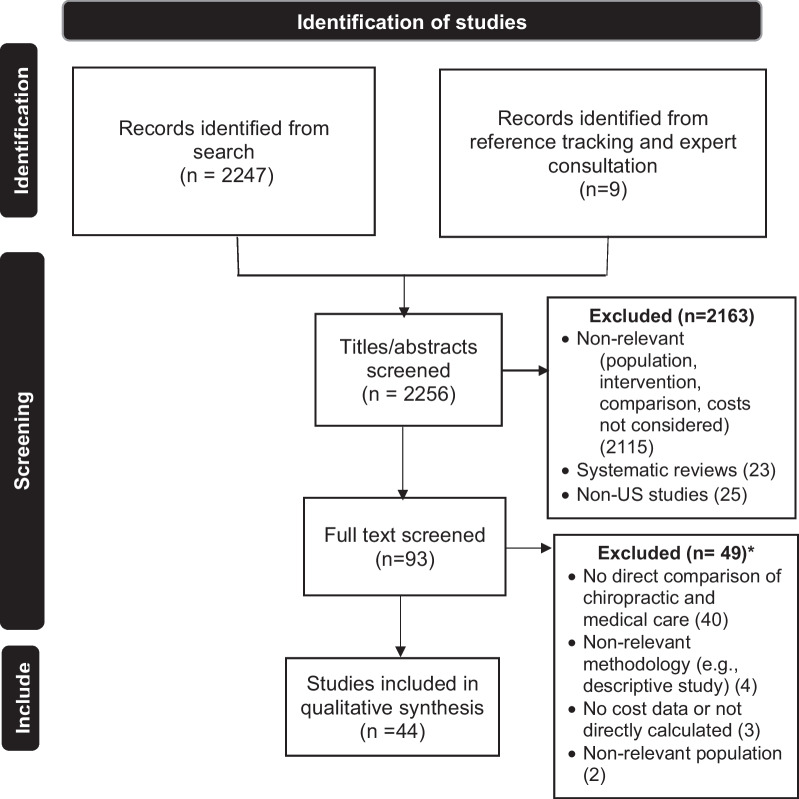
PRISMA (Preferred Reporting Items for Systematic Reviews and Meta-Analyses) flow diagram of literature search. *Excluded studies are listed in Supplementary materials

We concluded the search in November 2022 and retrieved 2247 citations. Figure 1 shows the PRISMA flow chart, showing reasons for exclusions. Nine articles were identified by reference tracking and expert consultation to make the total number of articles for screening 2256. Title and abstract screening of these resulted in 93 articles for full-text screening, with 49 excluded (see Additional File 2 for citations) and 44 remaining for quality assessment and data extraction. Please refer to Table 5 for a summary of findings including a quick-view color coded identification format related to each accepted paper. For ease of viewing, we divided the papers using two headings: (1) types of costs and (2) factors affecting costs.

### Final inclusions and quality assessment

Table 1 lists the study design and quality rating for the 44 included studies. All were rated high or acceptable quality (see Additional File 3 for details for the quality assessment) [20]. There were 4 prospective cohort studies [23–26], 22 retrospective or cross-sectional cohort studies [9, 12, 27–46], 17 cost studies [5, 47–62] and one randomized controlled trial [63], although 2 of the cost studies used data from RCTs.

**Table 1:** Included studies, by study design and first author

References	Title	Year	Rating*
	*Prospective cohort studies*		
Carey [23]	The outcomes and costs of care for acute low back pain among patients seen by primary care practitioners, chiropractors, and orthopedic surgeons	1995	A
Elder [24]	Comparative effectiveness of usual care with or without chiropractic care in patients with recurrent musculoskeletal back and neck Pain	2018	A
Graves [25]	Factors associated with early magnetic resonance imaging utilization for acute occupational low back pain: a population-based study from Washington State workers' compensation	2012	A
Keeney [26]	Early predictors of lumbar spine surgery after occupational back injury: results from a prospective study of workers in Washington State	2013	A
	*Cohort studies (retrospective/cross-sectional)*		
Anderson [27]	Three patterns of spinal manipulative therapy for back pain and their association with imaging, injection procedures, and surgery: a cohort study of insurance claims	2021	A
Anderson [28]	Risk of treatment escalation in recipients vs nonrecipients of spinal manipulation for musculoskeletal cervical spine disorders: analysis of insurance claims	2021	H
Bezdjian [29]	Efficiency of primary spine care as compared to conventional primary care: a retrospective observational study at an Academic Medical Center	2022	H
Davis [30]	The effect of reduced access to chiropractic care on medical service use for spine conditions among older adults	2021	H
Davis [31]	Access to chiropractic care and the cost of spine conditions among older adults	2019	H
Fritz [32]	Importance of the type of provider seen to begin health care for a new episode low back pain: associations with future utilization costs	2016	H
Hong [33]	Clinician-level predictors for ordering low-value imaging	2017	H
Hurwitz [34]	Variations in patterns of utilization and charges for neck pain in North Carolina, 2000 to 2009: a statewide claims' data analysis	2016	H
Hurwitz [35]	Variations in patterns of utilization and charges for the care of low back pain in North Carolina, 2000 to 2009: a statewide claims' data analysis	2016	H
Jin [36]	Healthcare resource utilization in management of opioid-naive patients with newly diagnosed neck pain	2022	H
Kazis [37]	Observational retrospective study of the association of initial healthcare provider for new-onset low back pain with early and long-term opioid use	2019	H
Liliedahl [38]	Cost of care for common back pain conditions initiated with chiropractic doctor vs medical doctor/doctor of osteopathy as first physician: experience of one Tennessee-based general health insurer	2010	H
Louis [39]	Association of initial provider type on opioid fills for individuals with neck pain	2020	H
Rhon [12]	The influence of a guideline-concordant stepped care approach on downstream healthcare utilization in pts with spine and shoulder pain	2019	H
Weeks [40]	Cross-sectional analysis of per capita supply of doctors of chiropractic and opioid use in younger Medicare beneficiaries	2016	H
Weeks [41]	The association between use of chiropractic care and costs of care among older Medicare patients with chronic low back pain and multiple comorbidities	2016	H
Whedon [9]	Initial choice of spinal manipulation reduces escalation of care for chronic low back pain among older Medicare beneficiaries	2022	H
Whedon [42]	Long-Term Medicare Costs Associated With Opioid Analgesic Therapy vs Spinal Manipulative Therapy for Chronic Low Back Pain in a Cohort of Older Adults	2021	H
Whedon [43]	Initial choice of spinal manipulative therapy for treatment of chronic low back pain leads to reduced long-term risk of adverse drug events among older Medicare beneficiaries	2021	H
Whedon [44]	Association between utilization of chiropractic services for treatment of low back pain and use of prescription opioids	2018	H
Whedon [45]	Impact of chiropractic care on use of prescription opioids in patients with spinal pain	2020	H
Whedon [40]	Association between chiropractic care and use of prescription opioids among older Medicare beneficiaries with spinal pain: a retrospective observational study	2022	H
	*Cost studies*		
Grieves [47]	Cost minimization analysis of low back pain claims data for chiropractic vs medicine in a managed care organization	2009	A
Haas [48]	Cost-effectiveness of medical and chiropractic care for acute and chronic low back pain	2005	H
Harwood [5]	Where to start? A two-stage residual inclusion approach to estimating influence of the initial provider on healthcare utilization and costs for low back pain in the US	2022	H
Jarvis [49]	Cost per case comparison of back injury claims of chiropractic versus medical management for conditions with identical diagnostic codes	1991	A
Kominski [50]	Economic evaluation of four treatments for low-back pain: results from a randomized controlled trial	2005	A
Legorreta [51]	Comparative analysis of individuals with and without chiropractic coverage: patient characteristics, utilization, and costs	2004	H
Leininger [52]	Cost-effectiveness of spinal manipulative therapy, supervised exercise, and home exercise for older adults with chronic neck pain	2016	H
Mosley [53]	Cost effectiveness of chiropractic care in a managed care setting	1996	A
Nelson [54]	Effects of a managed chiropractic benefit on the use of specific diagnostic and therapeutic procedures in the treatment of low back and neck pain	2005	A
Phelan [55]	An evaluation of medical and chiropractic provider utilization and costs: treating injured workers in North Carolina	2004	A
Shekelle [56]	Comparing the costs between provider types of episodes of back pain care	1995	A
Smith [57]	Costs and recurrences of chiropractic and medical episodes of low-back care	1997	H
Stano [58]	A comparison of healthcare costs for chiropractic and medical patients	1993	A
Stano [59]	The economic role of chiropractic: an episode analysis of relative insurance costs for low back care	1993	A
Stano [60]	Further analysis of healthcare costs for chiropractic and medical patients	1994	A
Stano [61]	Chiropractic and medical care costs of low back care: results from a practice-based observational study	2002	A
Stano [62]	Chiropractic and medical costs of low back care	1996	A
	*Randomized controlled trial*		
Cherkin(63)	A comparison of physical therapy, chiropractic manipulation, and provision of an educational booklet for the treatment of patients with low back pain	1998	A

^*^A, acceptable quality, moderate risk of bias; H, high quality, low risk of bias

### Data extraction and summary

Because of the large number of studies, we separated the data extraction into two tables, using 2017, the year the U.S. declared the opioid epidemic [13], as the dividing line. Table 2 displays data extracted from each included study published between 2018 and 2022. Table 3 displays data extracted from each included study published between 1991 and 2017.

**Table 2:** Summary of included studies 2018–2022

References	Year	Design	Data source	Sample	Intervention and comparison	Costs or other related factors measured	Time interval	Outcomes
Bezdjian [29]	2022	CO	Patient EHR	2692 adult patients with new dx of spine-related disorder	Primary Spine Care DC vs PCMD	Frequency of escalated care	6 mo	DC patients: Less likely to be hospitalized
						including ED visits, imaging, injections, hospitalizations, surgeries,		(OR = .47), fill opioid prescription (OR = .43), receive spinal injection
						specialist referrals and opioid prescriptions		(OR = .56), or visit specialist (OR = .48)
								Spinal diagnostic imaging
								DC, 8% vs. MD, 14%
Harwood [5]	2022	CS	2015–2016 Health Care Cost Institute (HCCI)	3,799,593 adults with new diagnosis of LBP	Cohorts formed by provider first seen for initial LBP diagnosis:	“Downstream” utilization of:	1-year post-LBP diagnosis	Opioid prescriptions
					1) AC	Opioids		Lowest for DC, AC or PT
					2) APRN	MRI, CT, radiography		Early prescription lower with AC or DC first and highest for EM or advanced practice RN
					3) DC	LBP surgery		Total cost lowest for DC ($5093) and PCMDs ($5660) first; highest for Ortho ($9434) or AC ($9205) first
					4) EM	ED visit		Out-of-pocket costs lowest for PCMD ($853) and DC ($911) first; highest for AC ($1415) and PM&R ($1238) first
					5) Ortho	In-patient hospitalization		· MRI/CT rate: 37%, 7% DC
					6) PM&R	Serious illness related to LBP		Beginning care with conservative provider resulted in significantly lower use of imaging and opioids
					7) PT	Total costs over 1 year		
					8) PCMD			
Jin [36]	2022	CO	IBM Watson Health MarketScan claims database 2007–2016	679,030 new-onset neck pain patients	Patients who did not receive early conservative care vs. those who did receive conservative care	Total healthcare costs, opioid use, healthcare service utilization (inpatient and outpatient)	1 year post-diagnosis	Early conservative therapy associated with 25% lower long term healthcare costs & with associated decreased opioid and ESI use
Whedon [9]	2022	CO	Medicare claims 2012–2016	28,160 MC beneficiaries with cLBP diagnosis	SMT vs OAT	Frequency of escalated care: hospitalizations, ED visits, advanced imaging, specialist visits,	5 years	Hospitalization:
						surgery, interventional pain med,		DC 1.4% MD 4.8%
						and encounters		Injections:
								DC 17%; MD 48%
								Adv imaging:
								DC 21%; MD 44%
								Specialist visit:
								DC 28%; MD 77%
								ED visit:
								DC 7%; MD 22%. Escalated care > 2.5 X higher for OAT vs SMT group
Whedon [46]	2022	CO	Medicare claims 2012–2016	55,949 MC beneficiaries	DC vs MD	Filling opioid prescription	1 year from initial visit	Risk for filling opioid prescription 56% lower for DC (hazard ratio 0.44)
				with spinal pain				
Anderson [27]	2021	CO	Insurance claims	10,372 unique back pain initial episodes	Initial SMT vs delayed SMT vs no SMT (medical care only)	Imaging, injections or back surgery	6 years	Initial SMT: 30% decrease in risk of imaging, injections or back surgery vs no SMT; risk with delayed SMT was higher than those with no SMT (22% Increase risk of escalation). I
			2012–2018					
Anderson [28]	2021	CO	Insurance claims	7951 unique neck pain initial episodes	SMT vs any care without SMT (PT included as “other care”)	Imaging, injections, emergency room, or surgery	6 years	Using SMT as reference (1.0), risks for other care:
			2012–2018					Imaging 1.8; injection 6.5; ED 16.9; surgery 7.3. Risk of escalation **2.1** for any group that did not receive SMT
Davis [30]	2021	CO	Medicare claims	39,278 MC chiropractic users	Use of medical services among chiropractic users who relocated and had decreased access to chiropractic vs those who did not	# of visits to PC MDs, surgeries, and overall costs for spine conditions	2 years before versus 2 years after relocation	Reduced DC access:
								Increased rate of PCMD visits for spine conditions
								Increased rate of spine surgeries
								Overall additional costs of medical services = $114,967 per 1,000 beneficiaries
Whedon [43]	2021	CO	Medicare claims	28,160 MC beneficiaries with long-term management of cLBP with SMT or OAT	SMT vs OAT	Adverse drug events (2)	12 months	Any ADE:
			2012–2016					SMT 0.9%; OAT 18.3%
								Opioid dependence/abuse:
								SMT 0.3%; OAT 14.3%
								ADE 51% lower in an outpatient setting with SMT. Long term care was 5X higher in OAT
Whedon [42]	2021	CO	Medicare Claims 2012–2016	28,160 MC with long-term care of cLBP with SMT or OAT	SMT vs OAT; Medical general and specialty practices, PM&R, DC, PT and Pain Management	Long-term total healthcare costs and LBP care costs	5 years	Mean LBP care long-term costs with OAT 58% lower than SMT
								Total long-term costs 1.87 times higher for OAT
Louis [39]	2020	CO	Marketscan research databases 2010–2014	427,966) patients with new-onset neck pain	Conservative (AC, DC, PT) vs PCP vs specialists (EM, Ortho, neurologists, PM&R, other)	Opioid prescriptions	Short term = 30 days after index visit; long term = 4 continuous quarters after index visit	AC had the lowest OR for opioid use; DCs had the lowest OR for opioid use at all time points compared to PT, PCP, Ortho, EM, PM&R, neurologist, and other. EM highest up to 90 days
Whedon [45]	2020	CO	Insurance claims 2012–2017	101,221 patients with spinal pain	SMT + PC MD vs no SMT, PC MD only	Opioid prescriptions	6 years	1.55 and 2.03 times more non-SMT patients filled an opioid prescription
Davis [31]	2019	CO	Medicare claims 2010–2014	84,679 MC chiropractic users who relocated	Use of medical services among chiropractic users with and/or neck pain who had decreased access to chiropractic vs those who did not	Cost of annual spine-related spending	**1** year	Higher spine-related spending on medical evaluation, management/procedures and diagnostic imaging and testing was associated with decreased access to chiropractic
Kazis [37]	2019	CO	OptumLabs Data Warehouse 2006–2015	216,504 new-onset LBP patients	Conservative (AC, DC, PT) vs specialist (PCP, Ortho, EM PM&R, MD-Other, neurosurgeon)	Opioid prescriptions	Short term = 30 days after index visit; long term = 4 continuous quarters after index visit	For both short and long -term prescriptions: Specialists had the highest OR and conservative (DC, PT, AC) the lowest
Rhon [12]	2019	CO	Military Health System (MHS) MHS Management and Reporting	7,566 patients with spine or shoulder pain	MT only vs MT + opioids; MT provided by PT, DO, or DC	total outpatient healthcare visits and costs, spine- and shoulder-related visits and costs, opioid prescriptions	1 year after index visit	All costs were lower for MT first
			Tool (M2) database					Costs, visits, and opioid prescriptions lower with:
								MT only
								MT early intervention before opioids (< 30 days from index)
Elder [24]	2018	PCO	EHR from Kaiser Permanente Northwest HMO	Sample size: 70 referred, 139 nonreferred patients	Standard care vs standard care + chiropractic	Clinical outcomes and costs of pain-related healthcare	2 years (2013–2015); patients followed up for 6 months	No statistically significant differences in either patient-reported
								or economic outcomes
Whedon [44]	2018	CO	NH administrative claims database 2013–2014	13,384 patients with primary LBP diagnosis	DC care vs non-DC care	Likelihood of opioid prescription fill; rate of prescription fill and associated costs	2 years	OR for opioid prescription fill was 0.45 for DC care with a 55% lower likelihood of filling an opioid prescription**;** opioid prescription costs were also significantly lower

Study designs: *CO* Retrospective/cross-sectional cohort study; *CS* Cost study/economic evaluation; *PCO* prospective cohort study

AC Acupuncturist; *ADE* Adverse drug event; *APRN* Advanced practice registered nurse; *cLBP* Chronic low back pain; *CT* Computed tomography; *DC* Chiropractor; *DO* Osteopathic physician; *ED* Emergency department; *EHR* Electronic healtth record; *EM* Emergency room medical physician; *LBP* Low back pain; *MC* Medicare; *MD* Medical doctor; *MRI* Magnetic resonance imaging; *MT* Manual therapy; *OAT* Opioid analgesic therapy; *OR* Odds ratio; *Ortho* Orthopedist/orthopedic surgeon; *PCP/PCMD* Primary care medical physician; *PM&R* Physical medicine and rehabilitation medical physician; *PT* Physical therapist; *RN* Registered nurse; *SMT* Spinal manipulative therapy

**Table 3:** Summary of included studies 1991–2017

References	Year	Design	Data source	Sample	Intervention and comparison	Costs measured	Time interval	Outcomes
Hong [33]	2017	CO	Insurance claims 2010–2014	878,720 adults with acute uncompli-cated back pain and 492,805 adults with acute uncompli-cated headache	100,977 clinicians, including PCMD vs DC vs specialist MD	Imaging	1 year	DCs did less imaging (17%) than specialists (36.5%) and more than PCMD (13.3%). DCs had higher Odds Ratio (OR) higher for imaging if they
								Owned X-ray equipment
								Had imaged prior patient
Hurwitz [34]	2016	CO	Blue Cross Blue Shield of NC claims by NC State Health Plan for Teachers and State Employees 2000–2009	2,795,046 UNP claims and 529,318 complicated neck pain CNP claims 2000–2009	DC alone, MD + PT, MD + DC, referrals (hospitals, emergency medicine, specialists, etc.)	Total cost of care for ICD9 diagnosis for one fiscal year	1 fiscal year	Costs excluding referral services: For UNP or CNP, MD + PT > MD + DC
								Costs including referral services: UNP or CNP: MD + PT > MD + DC
								UNP total charges: 54%-84% lower for DC
Hurwitz [35]	2016	CO	Blue Cross Blue Shield of NC claims by NC State Health Plan for Teachers and State Employees 2000–2009	2,075,866 ULBP claims and 1,083,496 CLBP claims 2000–2009	DC alone, MD + DC, MD + DC, referrals (hospitals, EM,specialists, etc.)	Total cost of care for ICD9 diagnosis for one fiscal year	1 fiscal year	Costs for ULBP:
								DC alone or MD + DC < MD alone or MD + PT
								Costs for CLBP:
								DC alone or MD + DC > MD alone or MD + PT
								Risk-adjusted:
								MD + DC < MD + PT and
								MD alone > DC alone for ULBP and CLBP
Weeks [41]	2016	CO	Medicare data 2006–2012	40,720 multiply comorbid patients aged 66 and older with cLBP episodes who were enrolled in Medicare Part D (56.3% of the total sample of 72,326)	1) CMT alone; 2) CMT followed by MD; 3) MD followed by CMT; 4) MD alone	Cost of care including pain medications	per episode costs	Costs and episode length:
								CMT alone < any other group
								CMT + MD < ,MD alone
Weeks [40]	2016	CO	Medicare data	Medicare patients in 2011 within the 306 Dartmouth hospital referral regions	Areas with higher and lower DC use by Medicare patients	Opioid prescriptions and Medicare payments to DCs	1 year	Higher DC costs (more usage) were associated with lower opioid prescriptions, but not with lower opioid doses in those with prescriptions
Leininger [52]	2016	CS using RCT data	RCT data	241 adults aged ≥ 65	Home exercise and advice (HEA) vs spinal manipulative therapy (SMT) plus HEA vs SRE plus HEA	Direct and indirect healthcare costs and clinical outcomes (pain, disability and QALY)	1 year	On average, SMT + HEA had better clinical outcomes and lower total societal costs than SRE + HEA and HEA alone, with a 0.75 to 0.81 probability of cost-effectiveness for willingness to pay thresholds of $50,000 to $200,000 per QALY
Fritz [32]	2015	CO	Claims data from University of Utah Health Plans 2012–2013	747 patients with new LBP claim	First provider	Number of:	1 year	DC first:
					1) Primary care MD 2) Physiatry	radiographs		Decreased risk for advanced imaging
					3) DC	Advanced imaging		Surgeon visit
					4) PT	Surgeon office visit		Increased episode of care duration
					5) Spine surgeon-Ortho/ neuro	Surgical procedure		
					6) ER	Epidural injection		
					7) Specialist	LBP-related EM		
						Costs: total allowed costs for all claims		
Keeney [26]	2013	PCO	Disability Risk Identification Study Cohort (D-RISC)	1885 WA state injured workers	First provider: DC vs. MD (occmed) vs MD (surgeon)	Early predictors of lumbar spine surgery, by type of provider	3 years	OR of surgery within 3 yrs: 1st provider-Surgeon 10.4; MD occmed 2.1; DC 0.2
								Surgery:
								43% of workers with surgeon first
								2% with DC first
Graves [25]	2012	PCO	Disability Risk Identification Study Cohort (D-RISC)	1830 WA state injured workers	First provider: DC vs MD (primary care) vs MD (occ med) vs MD (surgeon) vs ED vs other type (specialist or physical med)	Early predictors of early MRI, by type of provider	3 years (2002–2004); early MRI = lumbar MRI ≤ 42 days post injury	IRR (incident rate ratio):
								PCMD: 1.0
								DC: 0.6
								MD occ med: 1.4
								Surgeon: 1.5
								ED: 1.0
								Other: 1.2
								DC first:
								approximately 50% lower likelihood of early MRI as compared to PC MD
Liliedah l[38]	2010	CO	Blue Cross/Blue Shield TN records 2004–2006	85,402 patients with LBP	First provider: DC vs MD/DO	Cost of LBP care per episode(Total episode costs included costs paid for all services provided during the episode by any providers, including pharmaceuticals	By episode during the 2-year study period	Cost of episodes with initial DC, adjusted for risk, were 20% less than with initial MD
Grieves [47]	2009	CS	WI private HMO insurance claims database of ~ 30,000	Patients with at least 1 medical or chiropractic visit for LBP	Primary medical vs chiropractic vs specialist medical care	Mean total back pain claims for procedures by provider (DC or MD); medication costs not included	2 years	Per case, mean chiropractic cost was $851 and for all forms of medical care, $2784
			2004–2005	(n = 896)				Per case, median primary care medicine charges were $365; and $576 for all medical management; chiropractic $417 and specialist medical care $669
Haas [48]	2005	CS	Practice-based research network over 2-year period (1994–1996)	2872 patients with acute or chronic LB	Chiropractic care to primary medical care	Chart audit for a period of 12 months after baseline; office cost estimates based on Medicare/ ChiroCode relative value units and Medicare conversion factors. Estimated total costs included in-office costs plus estimated costs of advanced imaging, surgical consultation and physical therapist referrals	3 and 12 months from baseline visit	Adjusted DC office costs were 1.5–2.0 × greater than MD, but total costs of DC and MD treatment did not differ significantly at 3-months or 12-months when costs of advanced imaging and referrals are included
								Greater improvement in pain and disability with DC care vs MD care, without additional costs
Kominski [50]	2005	CS using RCT data	RCT data from records of a large medical practice treating HMO patients	681 patients with LBP	MD only, MD + PT, DC only, DC + PM	Total outpatient costs, excluding pharmaceuticals	18 mo	Adjusted mean outpatient costs: MD + PT $760
								DC + PM $579
								DC $560
								MD $369
Nelson [54]	2005	CS	Managed care insurance claims database from 1/1/97 through 3/30/01	Entire population of patients with chiropractic benefit (707,690) and without chiropractic benefit (1,001,995)	Insurance claims by patients for back or neck pain enrolled in medical plans with a chiropractic benefit vs those without a chiropractic benefit	Rates of advanced imaging, surgery, inpatient care, and plain-film radiographs	4-year	For patients with low back or neck pain use rates of all 4 studied procedures were significantly lower in the group with chiropractic coverage
Legorreta [51]	2004	CS	Administrative claims data from a large CA regional managed-care network	707,690 health plan members with an additional chiropractic coverage benefit; 1 M	Costs associated with episodes of care for patients with NMSK conditions receiving only DC care vs those receiving only MD care	Total healthcare claim costs, individual components of total costs (such as inpatient and outpatient services); costs of NMSK care at the episode level	4-year	Lower with DC care:
				without the chiropractic benefit				12% per member per year (PMPY) excluding medication costs
								13% PMPY costs with NMS patients
								Mean cost of DC back pain treatment was $522 (8% lower than patients without chiropractic)
								Complicated back pain episodes were only marginally higher (10% vs 8%) for MD vs DC care
								DC back pain patients had significantly fewer hospital days; lower MRI rate; lower surgery rate, fewer radiographs, and were less likely to have comorbidities
Phelan [55]	2004	CS	1975–1994, North Carolina Industrial	43,650 claims	Total cost of medical vs chiropractic management of injured workers in NC	Lost work days, Temporary Total Disability (TTD), MD cost, DC cost, hospital inpatient cost, hospital outpatient cost, total medical cost, compensation paid, and total cost of claim	All closed claims 1975–1994	LB injury: mean costs were $3425 for MD and $634 for DC. Compensation payments averaged $15,819 for patients with MDs, $1912 with DCs
			Commission closed injury claims					Mean lost workdays for MD care were 175; for DC care, 25. Mean total claim cost (including compensation) managed by MD was
								$23,562; for DC it was $2597. Note: There was only 0.8% DC utilization in this study, compared to 85.4% MD utilization
Stano [61]	2002	CS	Practice-based research network (1994–1996)	2872 patients with acute or chronic LB	Total cost of care for 922 medical patients vs cost of care for 1,950 chiropractic patients	Office visits and treatment utilizing CPT, RVU	1 year from initial visit	Mean office cost of DC care $214; MD non-referral care $103 (including prescriptions); with same degree of relief. Referral treatment, surgery, post-surgical care and advanced imaging costs excluded
Cherkin [63]	1998	RCT	RCT data from Group Health Cooperative of Puget Sound HMO	321 adults with LBP that persisted for 7 days after primary care visit	PT McKenzie method vs CMT vs provision of an educational booklet	Total costs to the HMO (no out-of-pocket expenses) including medications	Treated for 1 month; followed up for 2 years	2-year mean costs:
								PT $437
								$429 CMT
								$153 for the booklet group
								No significant differences in clinical outcomes
Smith [57]	1997	CS	MEDSTAT data from approximately 2 million beneficiaries	1215 patients (medical or chiropractic first)	Total cost and outcomes of medical vs chiropractic care for NMSK diagnoses	Total costs via total insurance and outpatient payments and patient retention	2 years	Total insurance payments greater for medically initiated episodes. Patients who "cross over" between providers are more likely to return to chiropractic providers
Mosley [53]	1996	CS	HMO data 1994–1995	121 chiropractic patients and 1838 medical care patients	Chiropractic vs medical patients with neck or back pain	Total cost of care including diagnosis, imaging, prescription meds,	1 year	Overall costs per patient: chiropractic = $539 vs medical = $774
								Imaging rate: chiropractic 5% vs 17% and cost/patient $31 vs $94
								# of prescriptions/pt: chiropractic 1 vs 2, Rx avg cost: Chiropractic-$3.25, Medical = $7.20
Stano [62]	1996	CS	MEDSTAT data from approximately 2 million beneficiaries	6183 patients (medical or chiropractic first)	Chiropractic vs medical patients with NMSK diagnoses	Total costs and episodes	2 years	Mean total payments for first episodes: Chiropractic $518 vs $1020
								Episode length: Chiropractic: 37 days vs 19 days
Shekelle [56]	1995	CS	RAND Health Insurance Experiment	686 patients	Chiropractic vs various types of medical care for patients with back pain (general practitioners, orthopedists, internist, DO, and all others.)	Number of visits per episode and mean costs per visit; total costs of episodes by provider type	4 wks before 1st visit to 4 wks after last visit	Mean provider cost/episode:
								DC $264; Ortho $247; DO $238; PC MD $95. Mean costs per visit:
								DC $19.45; PC MD $20.21; orthopedist $38.53, DO $22.18
Carey [23]	1995	PCO	Practice-based research network in NC	1633 patients with acute LBP	DC vs MD PC vs orthopedic surgeon	Total cost per episode of LBP (ambulatory)	24 weeks	Median costs/episode (urban):
								DC $545
								PCMD $169
								Surgeon $383
Stano [60]	1994	CS	MEDSTAT claims data from 395,641 patients with neuromusculoskeletal conditions.1988–1990	Patients receiving only medical/DO care; only chiropractic care; or both	Chiropractic plus medical/DO care vs medical/DO care only for patients with NMSK diagnoses	Total costs and hospital admission rates	2 years	Overall lower costs for patients using both chiropractic and medical care are attributable to lower rate of hospital admissions. Total cost outcomes: DC only = $4379, MD only = $5360
								Other spinal diagnoses also showed similar lower DC cost
Stano [59]	1993	CS	MEDSTAT data from approximately 2 million beneficiaries; 1988–1990	8928 patients with low back conditions with insurance that did not restrict chiropractic	Chiropractic vs medical/DO patients with LBP diagnoses	Total costs and episodes	2 years	Mean total payments:
								Chiropractic $573 vs $1112
								Episode length:
								Chiropractic: 39 days vs 22 days
Stano [58]	1993	CS	MEDSTAT claims data from 395,641 patients with neuromusculoskeletal conditions	Patients receiving only medical care; only chiropractic care; or both	Chiropractic plus medical care vs medical care only for patients with NMSK diagnoses	Total costs and hospital admission rates	2 years	Lower costs for patients using both chiropractic and medical care are attributable to lower rate of hospital admissions
Jarvis [49]	1991	CS	Workers Compensation claims for UT 1986	3062 workers with back injury claims treated by either MD or DC	Chiropractic vs medical costs for workers with back injuries	Total cost per case of care and compensation	2 years	Mean cost of care: DC $527 vs MD $684
								Mean days of compensation: DC 2 vs MD 21
								Mean compensation:
								DC $68 vs MD $668

Study design: CO, retrospective or cross-sectional cohort study; CS, cost study; PCO, prospective cohort study; RCT, randomized controlled trial

*AC* Acupuncturist; *cLBP* Chronic low back pain; *CLBP* Complicated low back pain; *CMT* Chiropractic manipulative treatment; *CNP* Complicated neck pain; *DC* Chiropractor or chiropractic care; *DO* Osteopathic physician or osteopathic care; *ED* Emergency department; *EM* Emergency medicine; *HEA* Home exercise advice; *HMO* Health maintenance organization; *LBP* Low back pain; *MD* Medical physician or medical care; *MRI* Magnetic resonance imaging; *Neuro* Neurosurgeon; *NMSK* Neuromusculoskeletal; *Occmed* Occupational medicine; *OMT* Osteopathic manipulative therapy; *OR* Odds ratio; *Ortho* Orthopedist/orthopedic surgeon; *PCMD* primary care medical physician; *PM* Physical modalities; *PMPY* Per member per year; *PT* Physical therapist or physical therapy care; *QALY* Quality-adjusted Life Year; *SMT* Spinal manipulative therapy; *SRE* Supervised rehabilitative exercise; *ULBP* Uncomplicated low back pain; *UNP* Uncomplicated neck pain

There were 17 included articles published in the 5 years from 2018 to 2022 (approximately 3 articles per year). There were 27 included articles published in the 26 years from 1991 to 2017 (approximately 1 article per year). From 2018 to 2022, most [15] were retrospective/cross-sectional cohort studies, with 1 prospective cohort study and 1 economic/cost study. From 1991 to 2017, most [16] were economic/cost studies, with 7 retrospective/cross-sectional cohort studies, 3 prospective cohort studies and 1 randomized controlled trial.

### Summary of cost factors

Table 4 summarizes the findings of all included studies, by year of publication. Below we have grouped these by type of cost and factors affecting cost. Table 5 depicts chiropractic services versus comparisons in terms of higher, lower or no difference in association for each of the identified types of costs and downstream utilization of factors affecting costs. All of the included studies newer than 2009 demonstrated associations that favored chiropractic services in regard to lower costs and lower utilization of services.

**Table 4:** Summary of findings for chiropractic management vs medical management, by year of publication

	Publication year	Study design	Summary
Bezdjian [29]	2022	CO	DC trained in Primary Spine Care—decreased:
			Hospitalization
			Opioid prescription fill
			ESI
			Specialist referral
			Diagnostic imaging
			Surgery
Harwood [5]	2022	CS	DC as 1st provider—decreased:
			Opioid and early opioid prescriptions
			Total cost, but similar to PCMD
			Out-of-pocket costs, but similar to PCMD
			MRI/CT
			1st provider—significantly less imaging and opioids
Jin [36]	2022	CO	DC or PT as 1st provider—decreased:
			Long-term healthcare costs
			Use of ESI
			Long-term opioid use
Whedon [9]	2022	CO	DC care—decreased:
			Escalation of care
			Hospitalization
			ESI and other interventional procedures
			Advanced diagnostic imaging
			Specialist visit/referral
			ED visit
Whedon [46]	2022	CO	DC care—decreased:
			Likelihood of filling opioid prescription
Anderson [27]	2021	CO	DC 1st provider—decreased
			Diagnostic imaging
			ESI/injection procedures
			Surgery
Anderson [28]	2021	CO	DC care—decreased:
			Escalation of care:
			Imaging
			ESI/injection procedures
			ED
			Surgery
Davis [30]	2021	CO	DC care—decreased:
			PCP, specialists, and surgeon visits for spine conditions
			Spine surgery
Whedon [42]	2021	CO	DC care:
			Increased LBP care long-term costs
			Decreased total long-term costs
Whedon [43]	2021	CO	DC care—decreased:
			Adverse drug events
			Opioid dependence/abuse
			Long term care
Louis [39]	2020	CO	DC care—decreased:
			Opioid use
Whedon [45]	2020	CO	DC care—decreased:
			Risk of filling opioid prescription
Davis [31]	2019	CO	DC care—decreased:
			Spine-related medical procedures
			Diagnostic imaging and testing
Kazis [37]	2019	CO	DC 1st provider—decreased:
			Short and long-term opioid prescriptions
Rhon [12]	2019	CO	Manual therapy—decreased:
			All costs, visits, and opioid prescriptions
Elder [24]	2018	PC	DC care compared to usual care:
			No statistically significant differences in costs
Whedon [44]	2018	CO	DC care—decreased:
			Likelihood of filling opioid prescription and cost of opioids
Hong [33]	2017	CO	DC care:
			Utilization of low value diagnostic imaging slightly less than specialists but more than PCP
			Clinician ownership of imaging equipment was a predictor of low value utilization across clinician type
Fritz [32]	2015	CO	DC care:
			Decreased advanced imaging
			Decreased surgeon visits
			Increased duration of episode of care
Hurwitz [34]	2016	CO	DC care—decreased:
			Costs for uncomplicated or complicated neck pain
Hurwitz [35]	2016	CO	DC care—decreased:
			Costs and episode length for uncomplicated LBP
			Costs for complicated LBP when care involved referral providers or services
Weeks [40]	2016	CO	Higher DC costs (more usage) were associated with lower opioid prescriptions
Weeks [41]	2016	CS using RCT data	DC care for chronic LBP episodes—decreased:
			Overall costs of care
			Episode duration
			Cost per episode
Leininger [52]	2016	CS using RCT data	DC care
			Decreased advanced imaging
			Decreased surgeon visits
			Decreased total healthcare costs
			Decreased lost productivity costs
			Increased duration of episode of care
Keeney [26]	2013	PCO	DC 1st provider—decreased:
			Back surgery
Graves [25]	2012	CO	DC care—decreased:
			Cost of episodes
Lilliedahl [38]	2010	CS	DC 1st provider—decreased:
			Overall episode costs
Grieves [47]	2009	CS	DC care:
			Increased office costs
			Approximately equal total costs for DC and MD when excluding costs of advanced imaging and referrals
Haas [48]	2005	CS	DC care:
			Increased office costs when excluding referrals
			DC and MD costs not significantly different when including referrals
Kominski [50]	2005	CS using RCT data	Excluding pharmaceutical data, adjusted mean outpatient costs greater for MD with PT, followed by DC with modalities and DC alone; MD alone lowest cost
Nelson [54]	2005	CS	DC care—decreased:
			Advanced imaging
			Surgery
			Hospitalization
			Plain film imaging
Legorreta [51]	2004	CS	DC care—decreased:
			PMPY costs
			Hospital days
			MRI and other imaging
			Surgery
Phelan [55]	2004	CS	DC care—decreased:
			Mean costs low back injury
			Compensation payments
			Mean lost workdays
			Mean total claim cost (including compensation)
			Utilization of medical ancillary services
			Hospitalization costs
Stano [61]	2002	CS	DC care:
			Increased mean office costs, when excluding costs of referral treatment, surgery, post-surgical care and advanced imaging
Cherkin [63]	1998	RCT	DC and PT care (McKenzie only) approximately equal and higher than cost of booklet
Smith [57]	1997	CS	DC care—decreased:
			Total insurance payments
			Patients with recurrent episodes tend to return to DC care
Mosley [53]	1996	CS	DC care—decreased:
			Overall costs per patient
			Imaging rate and cost per patient
			Prescriptions and prescription costs per patient
Stano [62]	1996	CS	DC care:
			Decreased total payments for first episodes
			Increased episode length
Carey [23]	1995	PCO	DC care:
			Increased cost per episode
Shekelle [56]	1995	CS	DC care:
			Increased cost/episode
			Approximately equal costs per visit with PCMD
Stano [60]	1994	CS	DC care—decreased:
			Overall costs due to decreased hospitalization
Stano [59]	1993 episode analysis	CS	DC or PCP care—decreased:
			Hospital admissions
			DC care—decreased:
			Episode costs
Stano [58]	1993	CS	DC care—decreased:
			Healthcare costs
Jarvis [49]	1991	CS	DC care:
			Increased number of office visits/case
			Decreased work-time loss compensation
			Decreased total cost per case
			Decreased cost per office visit

*CT* Computer tomography; *DC* Chiropractor or chiropractic care; *LBP* Low back pain; *MD* Medical physician or medical care; *MRI* Magnetic resonance imaging; *PCP/PCMD* Primary care medical physician; *PMPY* Per member per year; *PT* Physical therapist or physical therapy care

**Table 5:**
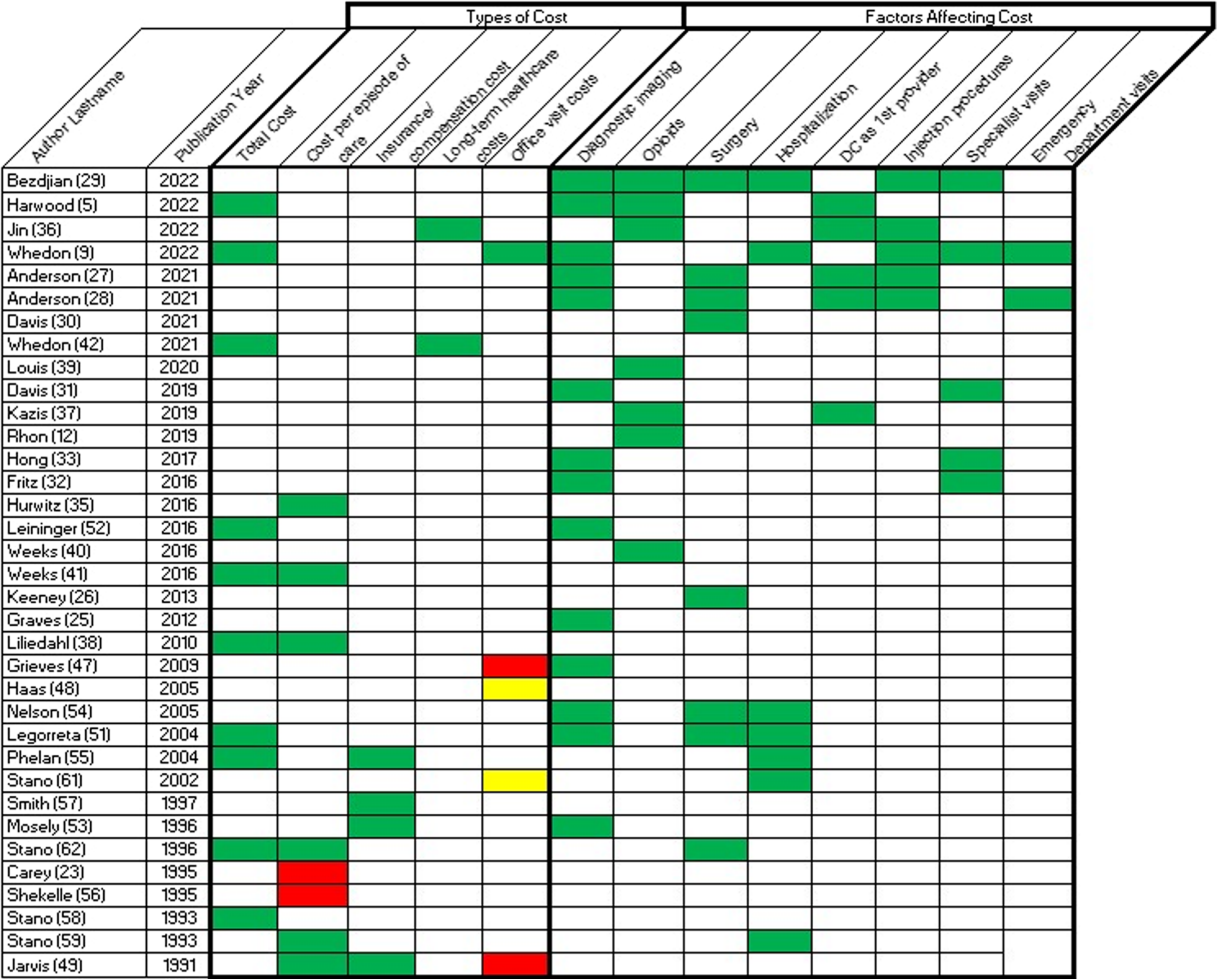
Association of chiropractic care with factors affecting costs, by study

Green = chiropractic associated with either lower cost *OR* lower utilization

Yellow = cost *OR* utilization did not significantly differ between groups

Red = chiropractic associated either higher cost *OR* higher utilization

White = study did not evaluate this cost type *OR* utilization

#### Type of costs

- *Total costs* Ten studies found that Doctor of Chiropractic (DC) care had lower overall costs [5, 12, 38, 41, 42, 51, 52, 55, 58, 62]. No studies found that DC care had higher overall costs.
- *Costs per episode of care* Six studies found that DC care had lower costs per episode [35, 38, 41, 49, 59, 62], and two found that it had higher cost per episode [23, 56].
- *Insurance/compensation costs* Four studies found DC care had lower insurance and compensation payment costs [49, 53, 55, 57]. No studies found higher costs.
- *Long-term healthcare costs* Two studies found lower long-term healthcare costs associated with DC care [36, 42]. No studies found higher costs.
- *Office visit costs* One study found reduced costs for DC office visits [12]; four studies found that DC care had higher costs for office visits [47–49, 61]. Two of these studies noted that chiropractic office costs were higher only when referral costs were not included in the calculation. When referral costs were included, chiropractic office visit costs did not differ significantly from medical care costs [48, 61].

#### Factors affecting costs

- *Diagnostic imaging* Fifteen studies found that diagnostic imaging, particularly advanced imaging like MRI, was used less with DC care; six studies were published 2018–2022 [5, 9, 27–29, 31] and nine studies from 1991 to 2017 [25, 32, 33, 47, 51–54, 61].
- *Opioids* Eleven studies found that fewer opioid prescriptions were dispensed or filled with DC care. Ten of these were published 2018–2022 [5, 12, 29, 36, 37, 39, 43–46], and only one between 1991 and 2017 [40].
- *Surgery* Eight studies found fewer surgeries with DC care; four published 2018–2022 [27–30] and 4 published 1991–2017 [26, 51, 54, 61].
- *Hospitalization* Seven studies found fewer hospitalizations with DC care; two studies were published from 2018 through 2022 [9, 29] and five from 1991 through 2017 [51, 54, 55, 59, 60].
- *DC as 1st provider* Six studies analyzed cost factors related to having a DC as the 1.^st^ care provider. Generally, this was associated with lower downstream costs. Four studies were published 2018–2022 [5, 27, 36, 37] and two published from 1991 through 2017 [26, 38].
- *Injection procedures* Five studies found decreased use of injection procedures with DC care; all were published from 2018 through 2022 [9, 27–29, 36].
- *Specialist visits (including surgeon referral visits)* Five studies found fewer referrals for specialist visits with DC care; three were published from 2018 through 2022 [9, 29, 31] and two published 1991–2017 [32, 33]. Three studies in the 1991–2017 group stated that their analyses had excluded all referral costs [47, 48, 61].
- *Emergency department (ED) visits* Two studies, both published from 2018 through 2022, found that fewer ED visits were associated with DC care [9, 28].

## Discussion

The purpose of this systematic review was to address our primary research question: Is chiropractic management of spine-related musculoskeletal pain in U.S. adults associated with lower overall healthcare costs as compared to medical care? This is the first systematic review of this type performed since 2015. In that review, Dagenais et al. found that health care costs were generally lower among patients whose spine pain was managed with chiropractic care. Due to the heterogeneity of patient populations and sample sizes each paper was evaluated by three separate reviewers using the checklists previously described in the Methods Sect. [15] As the literature review progressed, we found that in studies published within the past few years, an important aspect of cost began to emerge that went beyond the immediate per episode cost: the type of initial provider was strongly associated with lower downstream costs.

Downstream costs are often incurred after the initial provider has completed the episode of care. Downstream financial costs include expensive and invasive procedures such as hospitalization, surgery, injection procedures and advanced imaging. There are additional financial and non-financial downstream costs associated with the long-term consequences of addiction to opioid analgesics, including work absenteeism, decreased quality of life, psychological distress, and death due to drug overdose.

Bise et al. continued pursuing this concept in a 2023 cohort study, finding an association between the first choice of provider and future healthcare utilization [64]. His team concluded that both chiropractors and physical therapists provide nonpharmacologic and nonsurgical interventions, and that their early use appears to be associated with a decrease in immediate and long-term utilization of healthcare resources. This study adds further confidence in the emerging body of evidence on provider-related cost differentials and provides a compelling case for the influence of conservative care providers as the first provider managing for spine-related musculoskeletal pain. It follows logically that if downstream interventions are reduced, lower healthcare system costs will follow.

nonpharmacologic and nonsurgical interventions, and that their early use appears to be associated with a decrease in immediate and long-term utilization of healthcare resources. This study adds further confidence in the emerging body of evidence on provider-related cost differentials and provides a compelling case for the influence of conservative care providers as the first provider managing for spine-related musculoskeletal pain. It follows logically that if downstream interventions are reduced, lower healthcare system costs will follow.

The potential human and societal cost savings of avoiding overuse of opioid analgesics, with the possibility of overdoses and addiction, is another important emerging concept in the literature. We found that 10 studies published since the U.S. government declared the opioid epidemic in 2017 found reduced dispensing of opioid prescriptions when DCs were the first provider [5, 12, 29, 36, 37, 39, 43–46]. Only one study published in the earlier time period (1991–2017) included opioid prescribing as a comparison [41].

Overall, as summarized in Table 4, diagnostic imaging, opioid utilization, surgery, hospitalizations, injection procedures, specialist visits and emergency department visits were all reduced where chiropractors were involved early in the case. We did not subcategorize the patient populations (e.g., general population, Medicare, insurance claims) within any of tables.

## Limitations of the study

First, most of the included studies were retrospective cohort studies using large databases. Observational studies can only show associations, not prove causation, so definitive conclusions cannot be made about costs. However, their findings were so consistent that they warrant further scrutiny using higher-level study designs. Second, most of the included studies were retrospective cohort studies and therefore not the highest level of evidence. Third, we did not use any single validated checklist to assess study quality. We evaluated several checklists (e.g., SIGN, CHESS, MMAT) before determining that modification of validated checklists was necessary. Existing checklists seemed better-suited to prospective cohort designs and not as well-suited to the included retrospective cohort designs. The included studies were so variable in design and patient populations that it was not possible to pool the results for meta-analysis. Fourth, some large established datasets contained limited cost outcome variables. This made important factors such as pharmaceutical use and costs unavailable if they were not included in the dataset. Fifth, some observational studies using claims data and electronic health records do not provide enough detailed clinical information to determine whether opioid prescriptions were filled, or if filled, were actually used by the patient. Lastly, we searched only the MEDLINE database, and it is possible we missed other relevant articles not indexed there.

## Strengths

Although there are few randomized controlled trials available on this topic, there were many well-conducted cohort studies that provided analyses of large datasets with cost and care data identified by provider type.

While there are certainly other factors affecting cost of care, this paper included the most common cost escalators associated with typical care for LBP, including opioids, injection procedures, surgery, specialist visits and emergency department visits.

Based on the substantial body of evidence published since 1991, a trend is developing in US healthcare systems to include chiropractors as an integral part of the medical/healthcare team, as exemplified by the Veterans Administration (VA) chiropractic clinics across the country [65, 66].

**Recommendations.** When considering this evidence, it may be in society’s best interest for U.S. healthcare organizations and governmental agencies to consider modifying benefit designs to reduce barriers to access to chiropractic providers. Modifying or eliminating pre-authorization requirements, medical doctor gatekeepers, arbitrary visit limits, co-pays and deductibles may all be considered. Eliminating these barriers would allow easier access to chiropractic services, which based on currently available evidence consistently demonstrate reduced downstream services and associated costs.

## Conclusion

Patients with spine-related musculoskeletal pain who consulted a chiropractor as their initial provider incurred substantially decreased downstream healthcare services and associated costs, resulting in lower overall healthcare costs compared with medical management. A primary limitation was related to the heterogeneity and sample sizes of the populations and retrospective data sets. While observational studies cannot prove causation, the recurrent theme of the data seems to support the utilization of chiropractors as the initial provider for an episode of spine-related musculoskeletal pain. Future studies using randomized designs will be helpful in clarifying and validating this trend.

### Supplementary Information

**Additional file 1**: Search Strategy

**Additional file 2**: Articles excluded after full-text screening

**Additional file 3**: Quality assessment details

## Data Availability

The datasets used during the current study are available from the corresponding author on reasonable request.
