# Supplementary material for: Cost of chiropractic versus medical management of adults with spine-related musculoskeletal pain: a systematic review
Source: Chiropr Man Therap. 2024 Mar 6;32:8. doi: 10.1186/s12998-024-00533-4 (PMC10918856; doi:10.1186/s12998-024-00533-4)
Supplement: Supplementary file 1 — Additional file 1: Search Strategy [file 12998_2024_533_MOESM1_ESM.docx]

**Additional File 1: Search Strategy**

Filters: English, date limited to end 2022/10/31

("Chiropractic" OR "Chiro Therap*" OR "Chirotherap*" OR "Chiropraticien" OR "Chiropractie" OR "Chiropractor*" OR "Chiropratique" OR "Chiropraxie" OR "Manipulation Rachidienne" OR "Manipulation Vertebrale" OR "Manipulative Therap*" OR "Physical Medicine" OR "Quiropractica" OR "Spinal Manipulative Therap*" OR "Subluxation" OR "Electrotherapy" OR "Electrostimulation" OR "Laser Therapy" OR "Myofascial Release" OR "MFR therap*" OR "Myotherap*" OR "Soft Tissue Mobilization" OR "Joint Manipulation" OR "Musculoskeletal Manipulation" OR "Manipulative therap*" OR "Soft Tissue Manipulation" OR "Spinal Manipulation" OR "Manual Therap*" OR "Therapie Corporelle" OR "Therapie Manuelle" OR "Trigger Point Therap*" OR "Dry Needling" OR "Trigger Point Injection" OR "Trigger Point Management" OR “spinal adjustment” OR “physical therapy” OR “physical therapist” OR "Manipulation, Chiropractic"[Mesh] OR "Manipulation, Spinal"[Mesh])

AND

(cost OR costs OR "cost analysis" OR “cost benefit analysis” OR "Economics, Medical" OR "Insurance, health" OR "Fees and charges" OR "managed care programs" OR "Quality-adjusted Life Years" OR "cost effect*" OR "cost utility" OR "cost benefit" OR "cost minimiz*" OR "cost consequence" OR “cost comparison" OR “cost-efficien*” OR “cost-sav*” OR "economic evaluation" OR “health economics” OR insurance OR “insurance claim” OR “occupational back injury” OR “escalated care” OR “care escalation” OR "Managed Care Programs"[Mesh] OR "Cost-Benefit Analysis"[Mesh] OR "Costs and Cost Analysis"[Mesh] OR "Cost of Illness"[Mesh] OR "Health Care Costs"[Mesh] OR escalation OR “escalation of care” OR “downstream healthcare utilization” OR “downstream health care utilization” OR “treatment escalation”)

AND

(spine OR spinal OR neck OR disc OR intervertebral OR "back pain" OR "neck pain" OR cervical OR “low back” OR lumbar OR "Low Back Pain"[Mesh] OR "Neck Pain"[Mesh] OR "Pain Management"[Mesh] OR "Back Injuries"[Mesh])

NOT “spinal cord injur*”
