# Supplementary material for: Cost of chiropractic versus medical management of adults with spine-related musculoskeletal pain: a systematic review
Source: Chiropr Man Therap. 2024 Mar 6;32:8. doi: 10.1186/s12998-024-00533-4 (PMC10918856; doi:10.1186/s12998-024-00533-4)
Supplement: Supplementary file 2 — Additional file 2: Articles excluded after full-text screening [file 12998_2024_533_MOESM2_ESM.docx]

**Additional File 2: Articles excluded after full-text screening**

1. Butler RJ, Johnson WG. Adjusting rehabilitation costs and benefits for health capital: the case of low back occupational injuries. J Occup Rehabil. 2010;20(1):90-103.

2. Carey K, Ameli O, Garrity B, Rothendler J, Cabral H, McDonough C, et al. Health insurance design and conservative therapy for low back pain. Am J Manag Care. 2019;25(6):e182-e7.

3. Carey TS, Garrett J. Patterns of ordering diagnostic tests for patients with acute low back pain. The North Carolina Back Pain Project. Ann Intern Med. 1996;125(10):807-14.

4. Carey TS, Garrett JM, Jackman A, Hadler N. Recurrence and care seeking after acute back pain: results of a long-term follow-up study. North Carolina Back Pain Project. Med Care. 1999;37(2):157-64.

5. Castillo RC, Heins S, Feldman D, DuGoff EH, Roberts E, Staguhn ED, et al. The Impact of Adherence to Clinical Practice Guidelines on Medical Costs. J Occup Environ Med. 2020;62(9):712-7.

6. Chung SH, Bohl DD, Paul JT, Rihn JA, Harrop JS, Ghogawala Z, et al. Resource utilization for non-operative cervical radiculopathy: Management by surgeons versus non-surgeons. Clin Neurol Neurosurg. 2017;158:98-102.

7. Daffner SD, Hymanson HJ, Wang JC. Cost and use of conservative management of lumbar disc herniation before surgical discectomy. Spine J. 2010;10(6):463-8.

8. Davis MA, Martin BI, Coulter ID, Weeks WB. US spending on complementary and alternative medicine during 2002-08 plateaued, suggesting role in reformed health system. Health affairs (Project Hope). 2013;32(1):45-52.

9. Davis MA, Onega T, Weeks WB, Lurie JD. Where the United States spends its spine dollars: expenditures on different ambulatory services for the management of back and neck conditions. Spine. 2012;37(19):1693-701.

10. Deyo RA, Cherkin D, Conrad D, Volinn E. Cost, controversy, crisis: low back pain and the health of the public. Annu Rev Public Health. 1991;12:141-56.

11. Di Fabio RP, Mackey G, Holte JB. Disability and functional status in patients with low back pain receiving workers' compensation: a descriptive study with implications for the efficacy of physical therapy. Phys Ther. 1995;75(3):180-93.

12. Di Fabio RP, Mackey G, Holte JB. Physical therapy outcomes for patients receiving worker's compensation following treatment for herniated lumbar disc and mechanical low back pain syndrome. J Orthop Sports Phys Ther. 1996;23(3):180-7.

13. Emary PC, Brown AL, Cameron DF, Pessoa AF. Chiropractic integration within a community health centre: a cost description and partial analysis of cost-utility from the perspective of the institution. J Can Chiropr Assoc. 2019;63(2):64-79.

14. Gilkey D, Caddy L, Keefe T, Wahl G, Mobus R, Enebo B, et al. Colorado workers' compensation: medical vs chiropractic costs for the treatment of low back pain. J Chiropr Med. 2008;7(4):127-33.

15. Goldstone RA. Outcomes and costs of care for acute low back pain. N Engl J Med. 1996;334(5):329-30; author reply 30.

16. Graves JM, Fulton-Kehoe D, Jarvik JG, Franklin GM. Health care utilization and costs associated with adherence to clinical practice guidelines for early magnetic resonance imaging among workers with acute occupational low back pain. Health Serv Res. 2014;49(2):645-65.

17. Halfpap J, Riebel L, Tognoni A, Coller M, Sheu RG, Rosenthal MD. Improving Access and Decreasing Healthcare Utilization for Patients With Acute Spine Pain: Five-Year Results of a Direct Access Clinic. Mil Med. 2022.

18. Heins SE, Feldman DR, Bodycombe D, Wegener ST, Castillo RC. Early opioid prescription and risk of long-term opioid use among US workers with back and shoulder injuries: a retrospective cohort study. Inj Prev. 2016;22(3):211-5.

19. Herman PM, Whitley MD, Ryan GW, Hurwitz EL, Coulter ID. The impact of patient preferences and costs on the appropriateness of spinal manipulation and mobilization for chronic low back pain and chronic neck pain. BMC Musculoskelet Disord. 2019;20(1):519.

20. Heyward J, Jones CM, Compton WM, Lin DH, Losby JL, Murimi IB, et al. Coverage of Nonpharmacologic Treatments for Low Back Pain Among US Public and Private Insurers. JAMA Netw Open. 2018;1(6):e183044.

21. Huysmans E, Pien K, Callens L, Van Loon L, Ickmans K, Nijs J, et al. Determinants and Variations of Hospital Costs in Patients With Lumbar Radiculopathy Hospitalized for Spinal Surgery. Spine. 2019;44(5):355-62.

22. Ivanova JI, Birnbaum HG, Schiller M, Kantor E, Johnstone BM, Swindle RW. Real-world practice patterns, health-care utilization, and costs in patients with low back pain: the long road to guideline-concordant care. Spine J. 2011;11(7):622-32.

23. Jarvis KB, Phillips RB, Danielson C. Managed care preapproval and its effect on the cost of Utah worker compensation claims. J Manipulative Physiol Ther. 1997;20(6):372-6.

24. Kim CH, Chung CK, Choi Y, Lee J, Yang SH, Lee CH, et al. Direct medical costs after surgical or nonsurgical treatment for degenerative lumbar spinal disease: A nationwide matched cohort study with a 10-year follow-up. PLoS One. 2021;16(12):e0260460.

25. Krause CA, Kaspin L, Gorman KM, Miller RM. Value of chiropractic services at an on-site health center. J Occup Environ Med. 2012;54(8):917-21.

26. Kumar K, Malik S, Demeria D. Treatment of chronic pain with spinal cord stimulation versus alternative therapies: cost-effectiveness analysis. Neurosurgery. 2002;51(1):106-15; discussion 15-6.

27. Lee SW, Shen J, Kim SJ, Chun SY, Kim P, Riaz J, et al. US Trends of Opioid-use Disorders and Associated Factors Among Hospitalized Patients With Spinal Conditions and Treatment From 2005 to 2014. Spine. 2020;45(2):124-33.

28. Lewing B, Contreras J, Sansgiry SS. Demographics of and Costs to Users of Chiropractic Services in the United States with Neck or Back Pain not Meeting Guideline-Based Minimum Treatment Frequency Thresholds. Altern Ther Health Med. 2021.

29. McGowan JR, Suiter L. Cost-Efficiency and Effectiveness of Including Doctors of Chiropractic to Offer Treatment Under Medicaid: A Critical Appraisal of Missouri Inclusion of Chiropractic Under Missouri Medicaid. J Chiropr Humanit. 2019;26:31-52.

30. Mroz TM, Carlini AR, Archer KR, Wegener ST, Hoolachan JI, Stiers W, et al. Frequency and cost of claims by injury type from a state workers' compensation fund from 1998 through 2008. Arch Phys Med Rehabil. 2014;95(6):1048-54.e6.

31. Murphy DR, Justice B, Bise CG, Timko M, Stevans JM, Schneider MJ. The primary spine practitioner as a new role in healthcare systems in North America. Chiropr Man Therap. 2022;30(1):6.

32. Nahin RL, Stussman BJ, Herman PM. Out-Of-Pocket Expenditures on Complementary Health Approaches Associated With Painful Health Conditions in a Nationally Representative Adult Sample. J Pain. 2015;16(11):1147-62.

33. Nyiendo J. Disabling low back Oregon Workers' Compensation claims. Part II: Time loss. J Manipulative Physiol Ther. 1991;14(4):231-9.

34. Nyiendo J, Lamm L. Disabling low back Oregon workers' compensation claims. Part I: Methodology and clinical categorization of chiropractic and medical cases. J Manipulative Physiol Ther. 1991;14(3):177-84.

35. Scheer SJ, Radack KL, O'Brien DR, Jr. Randomized controlled trials in industrial low back pain relating to return to work. Part 1. Acute interventions. Arch Phys Med Rehabil. 1995;76(10):966-73.

36. Scheer SJ, Watanabe TK, Radack KL. Randomized controlled trials in industrial low back pain. Part 3. Subacute/chronic pain interventions. Arch Phys Med Rehabil. 1997;78(4):414-23.

37. Sears JM, Hogg-Johnson S, Sterling RA, Fulton-Kehoe D, Franklin GM. Prescription opioid overdose and adverse effect hospitalisations among injured workers in eight states (2010-2014). Occup Environ Med. 2020;77(7):439-45.

38. Sharma R, Haas M, Stano M, Spegman A, Gehring R. Determinants of costs and pain improvement for medical and chiropractic care of low back pain. J Manipulative Physiol Ther. 2009;32(4):252-61.

39. Spears CA, Hodges SE, Kiyani M, Yang Z, Edwards RM, Musick A, et al. Health Care Resource Utilization and Management of Chronic, Refractory Low Back Pain in the United States. Spine. 2020;45(20):E1333-e41.

40. Stason WB, Ritter GA, Martin T, Prottas J, Tompkins C, Shepard DS. Effects of Expanded Coverage for Chiropractic Services on Medicare Costs in a CMS Demonstration. PLoS One. 2016;11(2):e0147959.

41. Stephens B, Gross DP. The influence of a continuum of care model on the rehabilitation of compensation claimants with soft tissue disorders. Spine. 2007;32(25):2898-904.

42. Vavrek DA, Sharma R, Haas M. Cost analysis related to dose-response of spinal manipulative therapy for chronic low back pain: outcomes from a randomized controlled trial. J Manipulative Physiol Ther. 2014;37(5):300-11.

43. Walker J, Mertens UK, Schmidt CO, Chenot JF. Effect on healthcare utilization and costs of spinal manual therapy for acute low back pain in routine care: A propensity score matched cohort study. PLoS One. 2017;12(5):e0177255.

44. Wasiak R, McNeely E. Utilization and costs of chiropractic care for work-related low back injuries: do payment policies make a difference? Spine J. 2006;6(2):146-53.

45. Weeks WB, Goertz CM, Long CR, Meeker WC, Marchiori DM. Association Among Opioid Use, Treatment Preferences, and Perceptions of Physician Treatment Recommendations in Patients With Neck and Back Pain. J Manipulative Physiol Ther. 2018;41(3):175-80.

46. Whedon JM, Song Y, Davis MA. Trends in the use and cost of chiropractic spinal manipulation under Medicare Part B. Spine J. 2013;13(11):1449-54.

47. Whedon JM, Toler AWJ, Bezdjian S, Goehl JM, Russell R, Kazal LA, et al. Implementation of the Primary Spine Care Model in a Multi-Clinician Primary Care Setting: An Observational Cohort Study. J Manipulative Physiol Ther. 2020;43(7):667-74.

48. White PF, Elvir Lazo OL, Galeas L, Cao X. Use of electroanalgesia and laser therapies as alternatives to opioids for acute and chronic pain management. F1000Res. 2017;6:2161.

49. Williams DA, Feuerstein M, Durbin D, Pezzullo J. Health care and indemnity costs across the natural history of disability in occupational low back pain. Spine. 1998;23(21):2329-36.
