# Supplementary material for: Cost of chiropractic versus medical management of adults with spine-related musculoskeletal pain: a systematic review
Source: Chiropr Man Therap. 2024 Mar 6;32:8. doi: 10.1186/s12998-024-00533-4 (PMC10918856; doi:10.1186/s12998-024-00533-4)
Supplement: Supplementary file 3 — Additional file 3: Quality assessment details [file 12998_2024_533_MOESM3_ESM.docx]

**Additional file 3: Quality assessment details**

**Prospective cohort study quality assessment***

***See legend below this table. 1=Yes, 0=No, cannot determine or not reported**

| **First author, year** | **1** | **2** | **3** | **4** | **5** | **6** | **7** | **8** | **9** | **10** | **11** | **total** | **QA** |
| --- | --- | --- | --- | --- | --- | --- | --- | --- | --- | --- | --- | --- | --- |
| Carey(23) 1995 | 1 | 1 | 1 | 0 | 0 | 1 | 0 | 1 | 1 | 1 | 1 | 8 | A |
| Elder(24) 2018 | 1 | 1 | 1 | 0 | 1 | 1 | 0 | 1 | 0 | 1 | 1 | 8 | A |
| Graves(25) 2012 | 1 | 0 | 1 | 1 | 0 | 1 | 1 | 1 | 0 | 1 | 1 | 8 | A |
| Keeney(26) 2013 | 1 | 1 | 1 | 0 | 0 | 1 | 0 | 1 | 1 | 1 | 1 | 8 | A |

*QA= Quality assessment. Quality rating guide: 10-12= high quality, low risk of bias; 7-9=acceptable quality, moderate risk of bias; <7 =low quality, high risk of bias

|  | Item |
| --- | --- |
| 1 | Addresses an appropriate and clearly focused question. |
| 2 | Groups are similar at baseline except for factor of interest. |
| 3 | States number of people who accepted enrollment (response rate). |
| 4 | Analyses accounts for the likelihood that some patients might have the outcome when enrolled. |
| 5 | States attrition in each group and compares dropouts and compliant participants by exposure. |
| 6 | The outcomes are clearly defined. |
| 7 | Recognizes that knowledge of exposure status may affect outcome assessment. |
| 8 | Sources cited documenting validity and reliability of outcome assessment(s). |
| 9 | Identifies and accounts for main potential confounders in design and analysis. |
| 10 | Reports confidence intervals. |
| 11 | How well did the study minimize risk of bias or confounding?  Rate 2 for “excellent,” 1 for “acceptable,” 0 for “unacceptable.” |

**Retrospective and cross-sectional cohort studies quality assessment***

**Based on SIGN cohort checklist and NIH tool—website:** <https://www.nhlbi.nih.gov/health-topics/study-quality-assessment-tools>

***See legend below this table. 1=Yes, 0=No, cannot determine or not reported**

| **First author, year** | **1** | **2** | **3** | **4** | **5** | **6** | **7** | **8** | **9** | **10** | **11** | **total** | **QA** |
| --- | --- | --- | --- | --- | --- | --- | --- | --- | --- | --- | --- | --- | --- |
| Anderson(27) 2021 (LBP) | 1 | 0 | 1 | 0 | 1 | 1 | 1 | 1 | 1 | 1 | 1 | 9 | A |
| Anderson(28) 2021 (neck) | 1 | 1 | 1 | 1 | 1 | 1 | 1 | 0 | 1 | 1 | 2 | 11 | H |
| Bezdjian(29) 2022 | 1 | 1 | 1 | 1 | 1 | 1 | 1 | 0 | 1 | 1 | 2 | 11 | H |
| Davis(30) 2021 | 1 | 0 | 1 | 1 | 1 | 1 | 1 | 1 | 1 | 1 | 2 | 11 | H |
| Davis(31) 2019 | 1 | 0 | 1 | 1 | 1 | 1 | 1 | 1 | 0 | 1 | 2 | 10 | H |
| Fritz(32) 2016 | 1 | 1 | 1 | 0 | 1 | 1 | 1 | 1 | 1 | 1 | 2 | 11 | H |
| Hong(33) 2017 | 1 | 0 | 1 | 1 | 1 | 1 | 1 | 1 | 1 | 0 | 2 | 10 | H |
| Hurwitz(34) 2016 (neck) | 1 | 1 | 1 | 1 | 1 | 1 | 1 | 1 | 1 | 1 | 2 | 12 | H |
| Hurwitz(35) 2016 (LBP) | 1 | 1 | 1 | 1 | 1 | 1 | 1 | 1 | 1 | 1 | 2 | 12 | H |
| Jin(36) 2022 | 1 | 1 | 1 | 1 | 1 | 1 | 1 | 1 | 1 | 1 | 2 | 12 | H |
| Kazis(37) 2019 | 1 | 0 | 1 | 1 | 1 | 1 | 1 | 1 | 1 | 1 | 2 | 11 | H |
| Liliedahl(38) 2010 | 1 | 0 | 1 | 1 | 1 | 1 | 1 | 1 | 0 | 1 | 2 | 10 | H |
| Louis(39) 2020 | 1 | 0 | 1 | 1 | 1 | 1 | 1 | 1 | 1 | 1 | 1 | 10 | H |
| Rhon(12) 2019 | 1 | 0 | 1 | 1 | 1 | 1 | 1 | 1 | 1 | 0 | 2 | 10 | H |
| Weeks(40) 2016 (opioids) | 1 | 1 | 1 | 1 | 1 | 1 | 1 | 1 | 1 | 0 | 2 | 10 | H |
| Weeks(41) 2016 (MC costs) | 1 | 1 | 1 | 1 | 1 | 1 | 1 | 1 | 0 | 1 | 1 | 11 | H |
| Whedon(9) 2022 (initial choice) | 1 | 0 | 1 | 1 | 1 | 1 | 1 | 1 | 1 | 1 | 2 | 11 | H |
| Whedon(42) 2021 (MC/opioids) | 1 | 0 | 1 | 1 | 1 | 1 | 1 | 1 | 1 | 1 | 2 | 11 | H |
| Whedon(43) 2021 (MC AE) | 1 | 0 | 1 | 1 | 1 | 1 | 1 | 1 | 1 | 1 | 2 | 11 | H |
| Whedon(44) 2018 LBP/opioids | 1 | 0 | 1 | 1 | 1 | 1 | 1 | 1 | 1 | 1 | 2 | 11 | H |
| Whedon(45) 2020 | 1 | 0 | 1 | 1 | 1 | 1 | 1 | 1 | 1 | 1 | 2 | 11 | H |
| Whedon(46) 2022 (MC Opioids) | 1 | 0 | 1 | 1 | 1 | 1 | 1 | 1 | 1 | 1 | 2 | 11 | H |

*QA= Quality assessment. Quality rating guide: 10-12= high quality, low risk of bias; 7-9=acceptable quality, moderate risk of bias; <7 =low quality, high risk of bias

| 1. Research question or objective clearly stated? |
| --- |
| 2. Groups are similar at baseline except for factor of interest? |
| 3. Inclusion and exclusion criteria prespecified and uniformly applied? |
| 4. Exposure(s) of interest measured prior to the outcome(s) being measured? |
| 5. Timeframe sufficient to expect to see association between exposure and outcome? |
| 6. Outcomes are clearly defined. |
| 7. Exposures are measured appropriately in relation to outcome? |
| 8. Exposure measures (independent variables) and outcome measures (dependent variables) clearly defined, valid, reliable and implemented consistently? |
| 9. Reports confidence intervals? |
| 10.Key potential confounding variables measured and adjusted statistically for their impact on the relationship between exposure(s) and outcome(s)? |
| 11.How well did the study minimize risk of bias? (**2=excellent; 1=acceptable; 0=unacceptable)** |

**Cost study quality assessment.***

**Based on SIGN economic evaluation checklist**

See legend below this table. 1=Yes, 0=No, cannot determine or not reported

| First author, year | 1 | 2 | 3 | 4 | 5 | 6 | 7 | 8 | 9 | 10 | total | QA |
| --- | --- | --- | --- | --- | --- | --- | --- | --- | --- | --- | --- | --- |
| Grieves(47) 2009 | 1 | 1 | 1 | 1 | 1 | 0 | 0 | 0 | 0 | 2 | 7 | A |
| Haas(48) 2005 | 1 | 1 | 1 | 1 | 1 | 0 | 1 | 1 | 1 | 2 | 10 | H |
| Harwood(5) 2022 | 1 | 1 | 1 | 1 | 1 | 1 | 1 | 1 | 1 | 2 | 11 | H |
| Jarvis(49) 1991 | 1 | 1 | 1 | 1 | 1 | 0 | 0 | 0 | 1 | 1 | 7 | A |
| Kominski(50) 2005 | 1 | 1 | 1 | 1 | 1 | 0 | 0 | 0 | 1 | 1 | 7 | A |
| Legorreta(51) 2004 | 1 | 1 | 1 | 1 | 1 | 0 | 1 | 1 | 1 | 2 | 10 | H |
| Leininger(52) 2016 | 1 | 1 | 1 | 1 | 1 | 0 | 1 | 1 | 1 | 2 | 10 | H |
| Mosley(53) 1996 | 1 | 1 | 1 | 1 | 1 | 0 | 0 | 0 | 1 | 2 | 8 | A |
| Nelson(54) 2005 | 1 | 1 | 1 | 1 | 1 | 0 | 0 | 1 | 1 | 2 | 9 | A |
| Phelan(55) 2004 | 1 | 1 | 1 | 1 | 1 | 0 | 0 | 0 | 1 | 2 | 8 | A |
| Shekelle(56) 1995 | 1 | 1 | 1 | 1 | 1 | 0 | 0 | 0 | 1 | 1 | 7 | A |
| Smith(57) 1997 | 1 | 1 | 1 | 1 | 1 | 1 | 0 | 1 | 1 | 2 | 10 | H |
| Stano(58) 1993 | 1 | 1 | 1 | 1 | 1 | 0 | 0 | 0 | 1 | 1 | 7 | A |
| Stano(59) 1993 | 1 | 1 | 1 | 1 | 1 | 0 | 0 | 1 | 1 | 2 | 8 | A |
| Stano(60) 1994 | 1 | 1 | 1 | 0 | 1 | 0 | 0 | 1 | 1 | 1 | 7 | A |
| Stano(61) 2002 | 1 | 1 | 1 | 1 | 1 | 0 | 0 | 0 | 1 | 2 | 8 | A |
| Stano(62) 1996 | 1 | 1 | 1 | 1 | 1 | 0 | 0 | 0 | 1 | 2 | 8 | A |

*QA= Quality assessment. Quality rating guide: 10-11= high quality, low risk of bias; 7-9=acceptable quality, moderate risk of bias; <7 =low quality, high risk of bias

| 1. Addresses an appropriate and clearly focused question |
| --- |
| 1. Economic importance of the question is clear |
| 1. Choice of study design is justified |
| 1. All costs that are relevant from the viewpoint of the study are included and are measured and valued appropriately |
| 1. Outcome measures used to answer the study question are relevant to that purpose and are measured and valued appropriately |
| 1. If discounting of future costs and outcomes is necessary, it was performed correctly |
| 1. Assumptions are made explicit, and a sensitivity analysis performed |
| 1. The decision rule is made explicit, and comparisons are made on the basis of incremental costs and outcomes. |
| 1. The results provide information of relevance to policy makers. |
| 1. How well was the study conducted? (high quality=2; acceptable=1; unacceptable=0) |

**RCT quality assessment.***

Based on SIGN checklist

**See legend below this table. 1=Yes, 0=No, cannot determine or not reported**

| **First author, year** | **1** | **2** | **3** | **4** | **5** | **6** | **7** | **8** | **9** | **10** | **total** | **QA** |
| --- | --- | --- | --- | --- | --- | --- | --- | --- | --- | --- | --- | --- |
| Cherkin (63) 1998 | 1 | 1 | 1 | 0 | 0 | 1 | 1 | 1 | 1 | 1 | 8 | A |

*QA= Quality assessment. Quality rating guide: 9-10= high quality, low risk of bias; 6-8=acceptable quality, moderate risk of bias; <6 =low quality, high risk of bias

| 1. Addressed an appropriate and clearly focused question. |
| --- |
| 1. Random assignment to groups |
| 1. Power calculation used to justify sample size |
| 1. Investigators/assessors blinded to patients’ group assignment |
| 1. Patients blinded to group assignment |
| 1. Required sample size attained |
| 1. Sources cited documenting validity and reliability of outcome assessment(s) |
| 1. Groups similar in important characteristics at baseline, except factor of interest |
| 1. Attrition less than 20% |
| 1. Intention-to-treat analysis performed |
